# Stimulus-dependent modulation of perceptual and motor learning in a serial reaction time task

**DOI:** 10.2478/v10053-008-0112-2

**Published:** 2012-05-21

**Authors:** Waldemar Kirsch, Joachim Hoffmann

**Affiliations:** Department of Psychology, University of Würzburg, Germany

**Keywords:** serial reaction time task, sensorimotor learning, intermanual transfer

## Abstract

In two experiments, we investigated the impact of spatial attributes on the representation acquired during a serial reaction time task. Two sequences were used, in which structural regularities occurred either in the horizontal or in the vertical locations of successive stimuli. After training with the dominant hand, participants were required to respond with the non-dominant hand to either the original sequence or to a mirror-ordered version of the original sequence that required finger movements homologous to those used during training. We observed that a difference in reaction times between the two transfer conditions was smaller in the vertical sequence than in the horizontal sequence. This pattern of results was independent of whether three fingers (Experiment 1) were used or only one finger (Experiment 2) was used for responding. This result suggests that perceptual and motor learning mechanisms may be weighted differently depending on the context in which the stimulus is presented.

## Introduction

In serial reaction time (SRT) tasks, participants respond to sequences of stimuli with sequences of corresponding responses. Reaction times (RTs) typically decrease more quickly in response to structured sequences than to random sequences (Nissen & Bullemer, 1987), which suggests that participants acquire knowledge about the sequence structure. Another method often used to measure the learning of the sequence structure is to replace the structured sequence with a random sequence after participants have practiced a task. The magnitude of the decrease in performance in the random sequence may then reflect the magnitude of learning.

Despite much progress in this research area over the past few decades, the question of what people learn when producing movement sequences remains controversial. According to one view, performance benefits during structured sequences result because people learn the patterns of the stimulus sequences (Clegg, 2005; Cohen, Ivry, & Keele, 1990; Howard, Mutter, & Howard, 1992; Remillard, 2003). Other studies favor the view that learning is based on the structure of the response sequence (Hoffmann, Martin, & Schilling, 2003; Koch & Hoffmann, 2000a; Nattkemper & Prinz, 1997). Still others assert that learning is related to the sequence of response locations (e.g., Willingham, Wells, Farrell, & Stemwedel, 2000). Because diverse versions of the SRT task have been used, it is possible that these inconsistent findings are, to some extent, a product of task properties such as the types of stimuli or responses (cf. also De Roost & Soetens, 2006; Koch & Hoffmann, 2000b; Mayr, 1996; Richard, Clegg, & Seger, 2009).

The present study addressed this question by focusing on spatial attributes of the stimulus sequence. Specifically, we examined how horizontal and vertical regularities in the stimulus influence the amount of motor and perceptual knowledge of an individual. Several findings suggest that horizontally distributed visual stimuli are more effectively processed than vertically distributed stimuli. For instance, reading performance dramatically decreases when words are presented vertically (Bub & Lewine, 1988; Koriat & Norman, 1985; Lavidor, Babkoff, & Faust, 2001; Nazir & Huckauf, 2008; Rosazza, Cai, Minati, Paulignan, & Nazir, 2009). Moreover, when orientation angles become greater than 60°, reading time increases with word length. This finding suggests a switch from a parallel processing mode of letters to a serial processing mode (Koriat & Norman, 1985; Lavidor et al., 2001). A similar phenomenon has also been reported in the research of the so-called *crowding effect*, which occurs when a target becomes more difficult to perceive when it is embedded in adjacent distractors. Feng, Jiang, and He (2007) reported that a stronger crowding effect occurred when distractors were horizontally flanking the target than when distractors were vertically flanking the target. The authors assumed that a tendency to organize items into units may be more strongly pronounced for horizontally oriented spatial layouts than for vertically arranged items because of participants’ reading experience. According to Feng at al. (2007), the results may also reflect differences between horizontal and vertical dimensions in attentional resolution (cf. also Awh & Pashler, 2000). Furthermore, several studies have reported that perception across the visual field is not homogeneous at equal eccentricities. One well documented finding is referred to as *horizontal-vertical asymmetry*, which suggests that performance is better at isoeccentric spatial locations on the horizontal than on the vertical meridian (Carrasco, Evert, Chang, & Katz, 1995; Carrasco, Talgar, & Cameron, 2001; Rijsdijk, Kroon, & Van der Wildt, 1980). These studies suggest perceptual and attentional mechanisms within the visual system favor processing of horizontally distributed stimuli over the processing of vertically distributed stimuli.

Adhering to these findings one may assume that the spatial attributes of a sequence affect associative learning processes like those involved in SRT tasks. In particular, perceptual learning of successive stimuli may be more effective if the sequence structure is characterized by horizontal, rather than by vertical, regularities. This, however, does not need to be expressed in the overall performance of an SRT task because multiple aspects of the sequence structure, including other possible associations (e.g., of responses, response effects, or of response locations), can be acquired simultaneously (cf. Bapi, Doya, & Harner, 2000; Clegg, DiGirolamo, & Keele, 1998; De Roost, Zeeuws, & Soetens, 2006; Goschke, 1998; Hikosaka, Nakamura, Sakai, & Nakahara, 2002; Mayr, 1996; Seger, 1998; Verwey & Clegg, 2005; Witt & Willingham, 2006). For instance, according to the model of Hikosaka and colleagues (2002), spatial and motor sequence learning mechanisms operate in parallel, but they contribute differently to the task performance depending on the amount of practice. Spatial learning is assumed to dominate during initial learning, while motor learning largely supports long-term retention of a sequential skill. Similar differences in the dominance or weighting of a particular learning type might occur as a result of the layout of the spatial stimulus, which may either benefit or hinder a learning mechanism. Mayr (1996) demonstrated that sequence learning may be based on independent and parallel learning of sequences of objects and on sequences of stimulus locations. This finding indicates that certain learning mechanisms may prevail depending on the stimulus context. Moreover, a study by Koch and Hoffmann (2000b) asserted that learning may be determined by the availability of spatial features in the stimulus or response sequences. Learning was primarily based on the structure of the response sequence when the responses were spatially distributed (Experiment 3), while the stimulus sequence was learned only when the stimuli were spatially distributed (Experiment 2). These results suggest that if one learning form is li-mited by task context, then the other learning processes may dominate.

Against this background, we introduced conditions that selectively affected the relative salience of either the horizontal or the vertical stimulus dimension. A repetitive subsequence of three elements was embedded in a fixed order of nine two-dimensional spatial positions. The subsequence was exclusively related to the position order of the stimulus either on the horizontal or on the vertical dimension. Thus, we varied the relative amount of regularity (i.e., of redundancy) along the two dimensions while keeping all other stimulus properties constant. Two questions were examined in the present study. First, we sought to determine whether perceptual advantages of horizontal processing over vertical processing would enhance an individual’s ability to learn the perceptual structure of a sequence (i.e., stimulus-based or response location-based learning). In particular, we wanted to explore whether the learning of the sequence of two-dimensional positions of stimuli and/or of response keys might benefit if the horizontal location of each stimulus were highly predictable.^1^ Second, if such an effect were detectible, would response-based (i.e., motor) learning mechanisms contribute more substantially to sequence acquisition for stimuli that are less predictable on the horizontal dimension than on the vertical dimension? Assuming that stimulus context may hinder one learning mechanism, yet simultaneously facilitate other processes (see below), one might expect response-based learning mechanisms to receive more weight with vertical redundancies when perceptual learning is more difficult.

Perceptual and motor components of learning were accessed by means of intermanual transfer (cf. De Roost et al., 2006; Grafton, Hazeltine, & Ivry, 2002; Parasher, Roy, & Gordon, 2001; Verwey & Clegg, 2005). We followed a rationale that responding to the learned sequence of stimuli with the untrained hand would indicate perceptual learning. This was assumed because the sequence of effector movements is changed in this condition, whereas the sequence of stimuli and response keys remains unchanged (i.e., the parallel condition). The amount of motor learning was assumed to be expressed during the response to a mirrored version of the learned stimulus sequence, which involves effector movements homologous to those used during training (i.e., the mirror condition).^2^

## Experiment 1

Participants performed an SRT task, in which they responded to circular locations arranged in a 3 × 3 matrix by pressing assigned keys on a numerical keypad with their index, middle, and ring fingers. After an initial practice block, a fixed first-order conditional sequence of nine elements was repeatedly presented. The critical manipulation was related to the redundancies in the stimulus sequence. One group of participants practiced a sequence that could be parsed into three subsequences, each with three elements presented in the same succession of locations in the horizontal dimension (i.e., the horizontal sequence). That is, the order of right, left, and middle circle positions was repeated three times in the nine-element sequence. The second group of participants practiced another sequence, which was identical to the horizontal sequence in the statistical and the relational structures. However, it contained vertical regularities (i.e., the vertical sequence). After practicing with the dominant hand, participants had to perform the SRT task with their non-dominant hands. In one condition, participants responded to the original sequence of stimuli (and of response keys) with an unpracticed pattern of finger movements (parallel condition). In another condition, the stimulus sequence was modified to reverse the left and right targets around the vertical midline leading to the response sequence, which involved finger movements homologous to those used during training (mirror condition). Accordingly, participants had to rely on a sequence of homologous finger movements by responding to a changed stimulus sequence. As a consequence of more effective perceptual learning, we expected better intermanual transfer of the horizontal sequence, compared with the vertical, in the parallel condition. In the mirror condition, in contrast, the vertical sequence might be better transferred to the untrained hand than the horizontal sequence due to greater sequential motor knowledge.

### Method

#### Participants, task, and apparatus

Twenty-eight undergraduate students of the University of Würzburg participated in the study to partially fulfill their course requirements. They gave their informed consent to engage in the procedures. The sample was comprised of 17 females and 11 males between the ages of 19 to 28 years (*M*_age_ = 21.25). Twenty-six participants were predominantly right-handed and the remaining two were predominantly left-handed.

**Figure 1.**
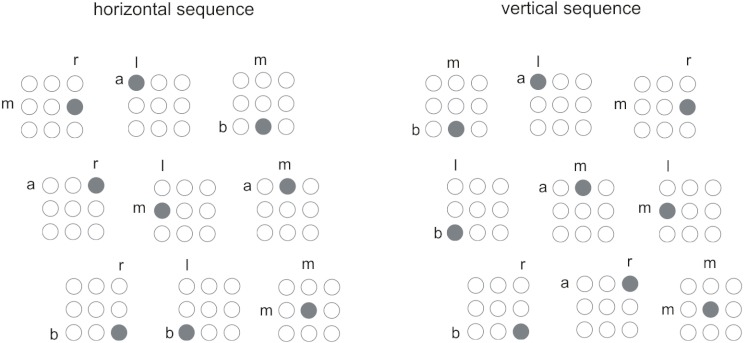
S equences of stimuli used in Experiment 1. Alphabetic characters indicate locations with respect to the horizontal and vertical dimensions: r = right, l = left, m = middle, a = above, b = below. The sequences are arranged from left to right and from top to bottom (i.e., the first element is top-left, the ninth is bottom-right).

Participants performed an SRT task. The visual stimuli consisted of nine grey circles arranged in a 3 × 3 array presented on a white background in the center of a 17-inch monitor. The viewing distance was approximately 50 cm. The circles were ~41 mm in diameter and were separated by ~60 mm (i.e., from center to center). In each trial, one of the nine circles was shaded to indicate the current stimulus location, and participants had to respond as quickly and accurately as possible to this stimulus (see Figure 1). Participants used the numerical keypad of a standard QWERTY keyboard to respond. The circle locations were compatibly assigned to the keys (i.e., the upper row of circles corresponded to the keys [7], [8], and [9], the middle row of circles corresponded to the keys [4], [5], and [6], and the lower row of circles corresponded to the keys [1], [2], and [3]). Participants were instructed to use their index, middle, and ring fingers when responding. The middle finger was aligned to the middle column, and the index and ring fingers were assigned to the outer columns. For instance, when the right hand was used, a participant responded to the circles appearing on the left side of the stimulus display by pressing the keys [1], [4], or [7] with their index finger, depending on the exact location of the stimulus.

#### Experimental procedure and design

The experiment consisted of 17 blocks, each consisting of 162 trials. Participants responded in the first 14 blocks using their dominant hand and in the last three intermanual transfer blocks with their nondominant hand. In the first, 12th, and 16th blocks, pseudo-random sequences of stimuli were presented to establish baseline blocks. These sequences were random with the constraint that the whole sequence of nine positions (i.e., filled circles) was completed before another repetition began. Moreover, immediate repetitions of stimuli were avoided to ensure high comparability with the regular sequences (see below).

Two nine-element first-order conditional sequences were used as the primary structured sequences in the remaining training blocks and completed using the dominant hand. These sequences were also completed in the intermanual transfer blocks using the nondominant hand. The Sequence Type constituted a between-subject factor. Fourteen participants were randomly assigned to one of two experimental groups.

The first group responded to the sequence shown in Figure 1 (left panel). Within this nine-element sequence, an additional structural redundancy was introduced by the threefold repetition of the stimulus location in the *horizontal dimension*. As shown in Figure 1, stimuli on the right side of the display were always followed by stimuli that appeared on the left side, which then triggered the middle display column. Thus, the sequence in the horizontal dimension could be parsed into three triplets of locations, which all contained a “right-left-middle” pattern of succession. In contrast, the order in the vertical dimension was more complex: “middle-above-below-above-middle-above-below-below-middle.”

The second group of participants was trained with a nine-element sequence, which was characterized by additional *vertical* regularities. In this group, the “below-above-middle” series was repeated three times, and the location succession in the horizontal dimension was complex (see Figure 1, right panel).

Both of these sequences were complementary because the order within and between horizontal triplets corresponded with the order of the vertical triplets. Moreover, they were also complimentary in the less relevant dimensions of both sequences (i.e., “middle-above-below-above-middle-above-below-below-middle” series corresponded to the “middle-left-right-left-middle-left-right-right-middle” series). Thus, this manipulation entailed sequences that had exactly the same statistical structure without differing in the relational structure (i.e., in respect to systematic relations within and between subsequences). The sequences only differed by the introduction of either vertical or horizontal dimensional redundancies.

During the intermanual transfer phase of the experiment, three different block types were presented. Participants responded to the same sequence of stimuli that they had previously practiced (parallel transfer), to the mirrored version of the original sequence (mirror transfer), and to a pseudo-random sequence (see above). To avoid a possible influence of the mirrored transfer on the parallel transfer blocks (and vice versa) and possible serial position effects, the succession of these three blocks was arranged to ensure the random block always occurred between the other two block types. This order of blocks was counterbalanced across participants.

The latency between the onset of the stimulus presentation and the key stroke was defined as the RT. As soon as the participant pressed a key, the next stimulus was presented. The refresh rate of the monitor was approximately 100 Hz; thus, a response-stimulus interval of ~10 ms was used. When a response was incorrect, the German word for *error* (“Fehler”) appeared on the monitor. Subjects received information about the mean RT of the previous responses at the end of each block. The RT difference between Blocks 13 and 14 was used as a measure of overall sequence learning, while the amount of intermanual transfer was assessed by comparing the performance in the last training block before the transfer phase (14) to the performance in the parallel and mirror transfer blocks.

To encourage subjects to follow the finger-key alignment, we asked them to press the center key with their middle fingers to start the blocks. Each regular block began at a randomly determined position in the nine-element sequence. After completing the SRT task, participants were debriefed about the presence and length of the sequence and were asked to recall the sequence. More specifically, they were asked to fill nine empty circle arrays by beginning at any position in the sequence. This recollection task was used as a test of sequence awareness.

### Results

**Figure 2.**
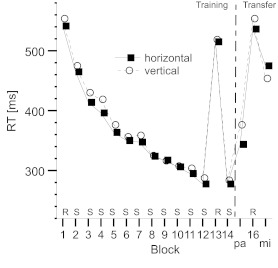
Sequences of stimuli used in Experiment 1. Alphabetic characters indicate locations with respect to the horizontal and vertical dimensions: r = right, l = left, m = middle, a = above, b = below. The sequences are arranged from left to right and from top to bottom (i.e., the first element is top-left, the ninth is bottom-right).

RTs from error trials (3.55 %) were excluded from the analysis. Moreover, responses that were more than three standard deviations above the mean RT, as determined separately for each participant and each block, were considered outliers and discarded from further analyses (1.93 % of responses). In the remaining trials, we computed median RTs for each subject and block of trials. The mean median RTs for each sequence and block are shown in Figure 2.

The initial levels of performance achieved in the first practice and in the next 11 training blocks were comparable in the horizontal and in the vertical conditions. We calculated the individual differences of RTs between Block 13 and Block 14 as a measure of overall structure-specific learning. The mean differences were 237 ms (*SD* = 59.48) for the horizontal sequence and 234 ms (*SD* = 61.60) for the vertical. These differences were significant, *t*(13) = 14.92, *p* < .001, and *t*(13) = 14.23, *p* < .001, and indicative of structure-specific learning. The difference between the two conditions was not significant, *t*(26) = 0.13, *p* = .902.

**Figure 3.**
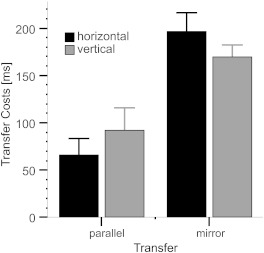
Mean intermanual transfer costs (reaction time differences between Block 14 and structured transfer blocks) as a function of the learned sequence and transfer type. Error bars represent standard errors.

To assess the completeness of the intermanual transfer, the RT differences between the intermanual transfer blocks, in which the original sequence (parallel condition) or its mirrored version (mirror condition) were presented, and the last training block (i.e., Block 14) were computed. An analysis of variance (ANOVA) performed on these transfer costs with the Sequence as the between-subjects factor and the Transfer Type as the within-subjects factor revealed a significant main effect of the transfer type, *F*(1, 26) = 94.62, *p* < .001, partial η^2^ =.784. More importantly, it revealed a significant Transfer Type × Sequence interaction, *F*(1, 26) = 6.23, *p* = .019, partial η^2^ = .193. As shown in Figure 3, the difference between the costs following the parallel transfer and the costs following the mirror transfer was significantly larger for the horizontal sequence (131 ms) compared with the vertical sequence (77 ms).

Additional analyses computed for each transfer condition, however, did not indicate significant differences between the two sequences for either the parallel transfer, *t*(26) = 0.88, *p* = .387, or the mirror transfer, *t*(26) = 1.18, *p* = .251.

We scored participants’ performance in the post-experimental recall task by determining the maximum number of sequence elements that were reproduced in the correct order. The horizontal sequence was associated with a mean of 5.4 correctly reproduced sequence elements, while 4.9 positions were recalled in the vertical sequence condition, *t*(26) = 0.53, *p* = .602. To test whether awareness of the sequence affected the observed results (cf. Willingham et al., 2000) we repeated the main analysis of the intermanual transfer costs for the subsample of participants who displayed only fragmented explicit sequence knowledge ( four elements) and for participants with more explicit knowledge (> four elements)^3^. In both groups, we obtained the same pattern of results as for the entire sample. However, the Transfer Type × Sequence interaction did not reach the threshold of significance for the implicit group, *F*(1, 14) = 0.88, *p* = .364, partial η^2^ = .059, but it was significant for the explicit group, *F*(1, 10) = 7.31, *p* = .022, η^2^ = .422.

### Discussion

Performance was generally better for the parallel transfer than for the mirror transfer. This result supports findings from several previous reports (e.g., Grafton et al., 2002; Verwey & Clegg, 2005) and indicates that the sequence of stimuli and/or of response locations may have contributed to sequence learning more than the sequence of effectors. However, the manipulation of horizontal versus vertical regularities modified the performance in both transfer conditions. The difference between the parallel and mirror transfer costs was smaller for the vertical sequence than for the horizontal sequence. This observed interaction is in accordance with our predictions and may suggest that a perceptual component was more strongly pronounced in the sequence knowledge when the horizontal stimulus dimension was accentuated. A motor component, in contrast, may have been weighted more heavily when the vertical dimension was more obvious.

Simple effect tests did not reveal significant results, and the observed pattern of results proved to be more pronounced in participants who possessed considerable explicit knowledge; thus, further research is necessary to better evaluate possible conclusions. One possible reason for the observed interaction may be related to the setup used in Experiment 1. Specifically, using three fingers to respond, as well as the applied key-finger assignment, may limit the validity and generalizability of the results. For instance, using three fingers may reinforce the relative salience of the horizontal dimension; this bias may be strengthened by the horizontal regularities. The assignment of the fingers to the three columns of the keypad may also differentially impact performance in the parallel and mirror transfer conditions depending on the sequence type. Because movement trajectories of each finger are more compatible with the regularities of the vertical sequence than with those of the horizontal sequence, the vertical condition may be associated with a stronger bias towards response-based learning. Furthermore, the relation between the introduced redundancies and the finger succession is not equal in both sequence conditions. With the horizontal sequence, the “right-left-middle” succession in the stimulus sequence corresponds to the succession “ring finger-index finger-middle finger”. No such relation is evident for the vertical sequence. Finally, Richard and colleagues (2009) recently used a variant of the SRT task, in which a sequence of alternating directions was embedded in the stimuli. This variant did not produce any repeating patterns in response locations. The sequence of directions was only learned when the subjects responded with their index fingers, which necessitated lateral arm movements between the response keys. In contrast, responding with four fingers, which did not require lateral movements, did not lead to sequence learning. This result suggests that different representations may be acquired depending on whether one or multiple effectors are used to respond.

## Experiment 2

In Experiment 2, we aimed to replicate and extend the results of Experiment 1. To evaluate the extent to which the use of multiple fingers and of a respective key-finger assignment may account for the pattern of results observed in Experiment 1, in Experiment 2 the participants were asked to respond with only their index fingers. All other manipulations remained the same as in the first experiment. If the difference between the parallel and mirror transfer costs were reduced for the vertical sequence, compared with the horizontal sequence, then this interaction would not be attributable to such specific factors as the number of effectors and the key-finger assignment.

### Method

The methods in Experiment 2 were nearly identical to those of Experiment 1, with the few differences described below.

Twenty-eight students of the University of Würzburg participated (25 women, three men; *M*_age_ = 23.32 years; age range of 19-39 years). None of these students had previously participated in Experiment 1. They provided their informed consent and received course credit at the end of the experimental session. Twenty-six participants were predominantly right-handed, and two participants were predominantly left-handed.

Participants performed the same SRT task as in Experiment 1. The only difference between the two experiments was in the effectors that were used. Instead of responding with three fingers as in the first experiment, participants were asked to react with only their index fingers. As a consequence of this change, there were no specific finger-keys assessments in this experiment.

### Results

**Figure 4.**
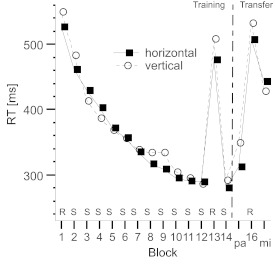
Mean reaction times (RTs) per block for the horizontal and vertical conditions in Experiment 2. Note that the order of parallel and mirror transfer blocks was counterbalanced across participants. R = random stimuli; S = sequenced stimuli; pa = parallel transfer; mi = mirror transfer.

RTs from error trials (2.80%) and outliers (2.23%) were excluded from analyses. The mean median RTs for the remaining trials are illustrated in Figure 4.

**Figure 5.**
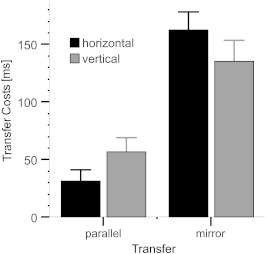
Mean intermanual transfer costs in Experiment 2. Error bars represent standard errors.

As shown in Figure 4, the performance pattern achieved with one finger in this experiment was very similar to that obtained with three fingers in Experiment 1. The regular structure was efficiently learned with the dominant hand in both sequence conditions, as indicated by the significant differences between the random block and the following structure block. Mean differences were 196 ms (*SD* = 68.11) for the horizontal sequence and 217 ms (*SD* = 72.79) for the vertical sequence; *t*(13) = 10.75, *p* <.001, for the horizontal condition; *t*(13) = 11.13, *p* < .001 for the vertical condition. The difference between the two conditions was not significant, *t*(26) = 0.79, *p* = .437. Moreover, we conducted an ANOVA to analyze the intermanual transfer costs. The Sequence Type served as the between-subjects variable, and Transfer Type served as the within-subjects factor, and the RT differences between Block 14 and the parallel and mirror transfer blocks were used. The results yielded a significant main effect of transfer type, *F*(1, 26) = 172.37, *p* < .001, partial η^2^ = .869, and a significant interaction between Transfer Type and Sequence Type, *F*(1, 26) = 10.46, *p* = .003, partial η^2^ = .287. As shown in Figure 5, the difference in transfer costs between the parallel and the mirror conditions was larger for the horizontal sequence (131 ms) compared with the vertical sequence (79 ms). However, as in Experiment 1, additional analyses computed separately for each transfer condition did not reveal significant differences between the two sequences, with *t*(26) = 1.56, *p* = .130, for the parallel transfer and *t*(26) = 1.12, *p* = .273, for the mirror transfer.

In the post-experimental recall task, the mean number of correctly reproduced sequence elements was 6.7 for the vertical sequence and 7.1 for the horizontal sequence, *t*(26) = 0.37, *p* = .711. We also repeated the main analysis of the intermanual transfer costs for two subsamples of participants, which were grouped by the median of elements that were correctly reproduced. Similar to the results of Experiment 1, a significant Transfer Type × Sequence interaction was present in participants with a high degree of explicit knowledge (> 8 elements), *F*(1, 11) = 16.09, *p* = .002, and η^2^ = .594. In contrast, the interaction was not significant in the group with less explicit knowledge ( 8 elements), *F*(1, 13) = 0.79, *p* = .389, and η^2^ = .057.

### Discussion

The results of Experiment 2 indicate that, as in Experiment 1, participants’ responses were slower in the mirror condition than in the parallel condition. More importantly, a significant Sequence Type × Transfer Type interaction was observed. The difference between the parallel and mirror transfer costs was smaller for the vertical sequence than for the horizontal sequence. This interaction pattern was also evident in participants with a high degree of explicit knowledge. Thus, the main results of Experiment 1 were replicated. Therefore, specific factors like the number of fingers or the finger-key assignment cannot account for the observed interaction between the dimension of spatial redundancies and the type of intermanual transfer.

## General discussion

We investigated the influence of visual stimulus characteristics on the nature of representations acquired during a perceptual-motor task in two experiments. The primary question of interest was whether the amount of motor and perceptual knowledge depends on spatial regularities in the horizontal dimension versus the vertical dimension. In one condition, the horizontal positions of stimuli were more predictable than the vertical positions (horizontal sequence). In another condition, the vertical stimulus dimension was more redundant (vertical sequence). We predicted a better intermanual transfer of the horizontal than of the vertical sequence in the parallel condition and predicted a reversed pattern in the mirror condition. We observed a difference in the intermanual transfer costs between the parallel and the mirror condition that depended on whether horizontal or vertical regularities were present in the stimulus sequence. This difference in transfer costs was smaller for the vertical sequence than for the horizontal sequence. However, we did not find significant differences between the two sequence conditions when the two transfer conditions were considered separately, although mean values indicated a trend in the expected direction. Thus, although the data allow only restricted conclusions, the results suggest that perceptual learning mechanisms may be more sensitive to the horizontal dimension of the sequence structure than to the vertical dimension and/or that motor learning may be more responsive to the vertical structure of a sequence.

The observed differences between parallel and mirror transfers for the horizontal sequence might be due to more effective processing of the horizontal stimulus features compared with the vertical stimulus features. As mentioned in the Introduction, there is evidence that perception may benefit more if stimuli are arranged along the horizontal meridian of the visual field than if they are arranged along the vertical meridian. Such a benefit for horizontal processing is not implausible because most of the relevant visual information related to our daily activities such as reading, walking, or driving is allocated along the horizontal dimension. Thus, as a result of ecological constraints, the visual system might be more strongly aligned with the horizontal than with the vertical dimension (cf. Carrasco et al., 2001). Structural and functional factors (such as attentional mechanisms, the structure of the visual field, and grouping processes) are discussed in this context, and these factors appear to account for the observed asymmetries in perception. These factors also appear to facilitate perceptual learning of horizontal regularities better than learning of vertical regularities. For instance, reading experience appears to be particularly relevant for the present task. It is possible that the learned tendency to organize horizontally arranged items into units led to more fluent processing and to stronger integration of single elements of the horizontal sequence compared with the vertical condition.

The observed difference between the two sequence conditions is also compatible with the assumption that in the vertical sequence, where perceptual learning might require more effort, subjects relied more heavily on effector movement sequences than in the horizontal condition. Because the overall learning performance was comparable in both sequence conditions, the results may suggest that subjects compensated for perceptual learning disadvantages by weighting motor information more heavily. This is expected if one assumes that multiple independent learning processes operate in parallel to optimize the performance and points to a high adaptivity of learning behavior.

The overall results provide evidence supporting the idea that spatial regularities affect the nature of representations acquired during perceptual-motor learning. Moreover, the results seem to align well with a number of previous reports that suggested task conditions may modulate learning mechanisms (De Roost & Soetens, 2006; Koch & Hoffmann, 2000b; Mayr, 1996; Richard et al., 2009). Our results also extend these findings by highlighting the flexibility of learning. They indicate that both perceptual and motor mechanisms may contribute to sequence acquisition and that the relative impact of distinct learning processes may depend on task conditions.

These conclusions are tentative and have to be considered with caution due to a number of factors that may limit functional interpretations. For instance, the performance in the mirror transfer condition may not necessarily reflect the amount of motor learning. According to the motor hypothesis, which served as our premise, activation of homologous movements may account for the mirror transfer. Such representations may operate on the level of hand postures (cf. Rosenbaum, Meulenbroek, & Vaughan, 1999); thus, the representations would be effector-specific. Alternatively, mirror performance may also be mediated by a transformation of the spatial representation of the learned stimulus sequence (Grafton et al., 2002; Verwey & Clegg, 2005). Al-though this question has not been examined in detail, the results do not support the spatial hypothesis. According to the spatial hypothesis, an advantage of the vertical sequence over the horizontal sequence would indicate a better spatial representation. However, this hypothesis seems implausible because the results of the parallel transfer condition, which captures spatial learning more directly, indicated an opposite pattern. Another possible weakness of the paradigm of intermanual transfer may also be related to the learning of the nondominant hand, because this learning may cause systematic sequence-specific RT biases that are independent of any prior practice with the dominant hand. However, because the overall learning performance of the dominant hand was comparable for both sequences, such an influence should not be expected.

Moreover, the observed differences in responding to stimuli with horizontal and vertical regularities may be relative rather than absolute. As suggested in the research of the spatial stimulus-response compatibility effects (e.g., Fitts & Seger, 1953), many results indicating a preference for horizontal coding over vertical coding (right-left prevalence effect) may be explained by a relative salience account (see Rubichi, Vu, Nicoletti, & Proctor, 2006, for a review). According to this research, coding takes place in the dimension that is made salient by the stimulus-response environment, and performance is best when the salient dimensions of the stimulus and response sets correspond. The overall prevalence of the horizontal dimension observed in many experiments (e.g., Nicoletti & Umiltŕ, 1984) arose because horizontal-salient response and/or stimulus configurations were used (see also Hommel, 1996). One may thus argue that the results of this study may be artifacts of the used setup. For instance, the correspondences between the introduced redundancies in the stimulus sequence and the resulting regularities in the response sequence were different for the vertical and the horizontal conditions. While the horizontal dimension in the stimulus sequence was compatible with the horizontal dimension in the response sequence (“right-left”), the vertical stimulus dimension (“above-below”) was related to the depth of the response sequence (“back-forth”). Consequently, the current results may have been affected by this incompatibility within the stimulus-response set that was used in this study.

Furthermore, we relied on a well-established assumption that local associations are formed between successive stimuli, successive responses, and/or successive response locations. Provided that this assumption is correct, the results of the mirror transfer condition are unambiguous. However, if more abstract perceptual and/or motor know-ledge has been acquired during the experiments (e.g., if subjects learned that all stimuli on the right side of the display are always followed by stimuli on the left side), then a possible benefit of the vertical sequence over the horizontal sequence in the mirror condition may be related to the differences in the fit of this knowledge to the features of the mirrored sequence. While the abstract regular structure of the vertical sequence is maintained after mirroring (i.e., the “below-above-middle” succession is also present in the mirrored version of the sequence), the learned “right-left-middle” succession of the horizontal sequence is not more present after mirroring. Thus, the greater difference between the parallel transfer and the mirror transfer for the horizontal sequence may be partially attributed to this loss of learning and, consequently, due to the impossibility of applying the mentioned type of knowledge on the new sequence.

It should also be mentioned that participants did not acquire significantly more explicit knowledge in the horizontal condition, which is associated with more perceptual learning. Perceptual learning is often assumed to be explicit, while motor learning is typically seen as implicit (cf. Hikosaka et al., 2002). We assume, however, that the implemented manipulation induced only minor differences in learning, so the post-experimental recall task was not able to capture these differences appropriately. In both experiments, the vertical sequences were associated with a mean number of correctly reproduced sequence elements, which was lower than the corresponding value in the horizontal condition. Comparatively, the overall performance in the recall task indicated a considerable amount of explicit knowledge, especially in Experiment 2. Moreover, the main pattern of results observed in both experiments was especially salient in subjects who possessed considerable amounts of explicit knowledge. This may indicate that the observed interaction occurred as a result of relatively high sequence awareness. This, in turn, may suggest that strategic, rather than automatic, mechanisms underlie the assumed interplay between perceptual and motor learning. The overall dominance of perceptual learning may also be related to participants’ high degrees of awareness of the sequence structure (cf. De Roost & Soetens, 2006).

Finally, it is unclear how eye movements may have affected the current results. A sequence of stimuli may be accompanied by a sequence of eye movements. Accordingly, learning may also be based on motor information of the ocular system, instead of, or in addition to, visual information (cf. De Roost & Soetens, 2006). If so, then measures derived from the parallel and mirror transfer conditions would include an ocular component, which may make an unambiguous distinction between perceptual and motor learning difficult.

To conclude, the results of this study suggest that the content of the memory trace generated in perceptual-motor tasks may vary depending on the context of the stimuli. Visual stimuli containing redundant information on the horizontal dimension appear to facilitate perceptual learning mechanisms. Vertical redundancies, in contrast, seem to enhance a motor component of learning. However, given the complexity of sensorimotor interactions and the relatively small size of the observed effects, further studies are needed to evaluate the validity and generality of these conclusions. Given some weaknesses in the method of intermanual transfer, other paradigms could be applied to replicate and extend the current results. For instance, a dissociation between stimulus-based and response location-based learning with another type of transfer task (cf. Willingham et al., 2000) may provide more detailed information about the mechanisms mediating a possible vertical-horizontal asymmetry in sequence learning tasks.

## Footnotes

1 However, we do not mean that a sequence of horizontal locations can be learned independently of a sequence of vertical locations. Horizontal and vertical characteristics of a stimulus cannot be distinguished in the present experiments because participants learned a sequence of two-dimensional positions. Nevertheless, we assume that perceptual and/or attentional factors might also facilitate learning (e.g., by a more rapid detection and/or grouping of single sequence elements) if only one of two dimensions of a sequence has salient attributes.

2 The terms *perceptual learning* and *motor learning* are operationally defined by the imposed transfer tests. In the parallel condition, participants see the same order of stimuli and press the keys in the same order as during acquisition. Due to the effector change, however, a new unpracticed pattern of muscle activation and joint angles has to be used in this test. Accordingly, measured performance can be considered indicative of stimulus-based and/or response location-based learning. In the mirror condition, in contrast, the motor coordinates are reinstated, while the visual coordinates of response locations and stimuli are altered. That is, participants have to respond with the same pattern of homologous muscle activation and use the same relative joint angles as during practice (cf. Kovacs, Han, & Shea, 2009). Accordingly, performance in the mirror test may be related to some aspects of motor learning, including the order of effectors or of effector movements.

3 The value of 4 corresponded to the median of the whole sample.
